# Prospective, Non-Blinded, Randomized Controlled Trial of Pulmonary Surfactant Administration Guided by Lung Ultrasound in Preterm Infants with Gestational Age < 32 Weeks

**DOI:** 10.3390/children12121618

**Published:** 2025-11-27

**Authors:** Jinghui Zhang, Jinfang Yuan, Jing Xu, Tongyan Han, Yahui Zhang, Huiqiang Liu, Danfang Lu, Yunfeng Liu

**Affiliations:** Department of Pediatrics, Peking University Third Hospital, Beijing 100191, China; penguin_post@sina.com (J.Z.); yuanjf9@126.com (J.Y.); xj33632@126.com (J.X.); tongyanhan@bjmu.edu.cn (T.H.); huiyaya326@163.com (Y.Z.); cclhq@126.com (H.L.); ludanfang2021@163.com (D.L.)

**Keywords:** lung ultrasound, scoring, pulmonary surfactant, respiratory distress syndrome, preterm infants, gestational age < 32 weeks

## Abstract

**Highlights:**

**What are the main findings?**

- Lung ultrasound-guided surfactant administration reduced invasive ventilation and radiation exposure in preterm infants.
- It enabled earlier and more precise PS use than FiO_2_-based methods.

**What are the implications of the main findings?**

- Lung ultrasound improves NRDS management and supports safer, evidence-based neonatal care.
- Incorporating lung ultrasound into clinical protocols may optimize resource use and guide updates to neonatal respiratory guidelines.

**Abstract:**

**Objectives**: Current guidelines for pulmonary surfactant (PS) administration in preterm infants with respiratory distress rely on clinical signs and FiO_2_ thresholds. Lung ultrasound offers a promising alternative for accurately diagnosing neonatal respiratory distress syndrome (NRDS) and assessing its severity. This randomized controlled trial aimed to evaluate whether a lung ultrasound-guided strategy for NRDS diagnosis and lung ultrasound scores (LUS)-guided PS administration could improve respiratory outcomes in preterm infants (<32 weeks’ gestation), compared to conventional methods. **Methods**: In this non-blinded randomized controlled trial, 89 preterm infants (≤32 weeks’ gestation) with respiratory distress after birth were enrolled. Participants were randomly assigned to either the ultrasound group (PS administration based on ultrasound-confirmed NRDS and LUS criteria) or the control group (PS administration according to standard clinical signs and FiO_2_ requirements). **Results**: The ultrasound group demonstrated a significantly lower rate of invasive mechanical ventilation (*p* = 0.007) and a shorter duration of ventilation (*p* = 0.005) compared to the control group. Furthermore, the ultrasound group required less PS (*p* = 0.03), received their first dose at an earlier time (*p* = 0.017), and experienced fewer radiation exposures both before surfactant treatment and within the first week after birth (*p* = 0.023 and *p* = 0.019, respectively). **Conclusions**: The integration of lung ultrasound for NRDS diagnosis and LUS-guided surfactant therapy facilitates more precise and timely PS use. This strategy reduces the need for and duration of invasive mechanical ventilation and limits early radiation exposure in very preterm infants.

## 1. Introduction

Neonatal respiratory distress syndrome (NRDS), primarily affecting preterm infants, results from pulmonary immaturity and a deficiency of pulmonary surfactant (PS) [1]. The cornerstone of its management includes continuous positive airway pressure (CPAP) and surfactant replacement therapy [2]. Current protocols recommend initiating CPAP in spontaneously breathing preterm neonates, with pre-emptive PS use guided by clinical evaluation of work of breathing and FiO_2_ [1]. This strategy, particularly when PS is administered within the “golden first hour,” is proven to lower mortality, decrease the incidence of bronchopulmonary dysplasia (BPD), and reduce the need for invasive ventilation [3,4]. The concept of the “golden first hour” further emphasizes the time-sensitive challenge for early PS use.

Nonetheless, applying these principles in practice presents challenges. The differential diagnosis for early respiratory distress extends beyond NRDS to include conditions like neonatal pneumonia, pulmonary edema, and meconium aspiration syndrome. Indeed, autopsy data suggest NRDS is misdiagnosed in up to 57% of cases [5]. Furthermore, not all infants with NRDS require PS; mild cases can often be managed with CPAP alone [1]. Furthermore, reliance on FiO_2_ thresholds is problematic due to its imperfect correlation with true oxygenation status and its potentially slow increase. These factors complicate clinical decision-making regarding early PS administration. Consequently, the question of whether early, widespread PS administration yields superior outcomes compared to a more targeted, diagnosis-driven approach remains unresolved [6]. The administration of surfactant, however, is not without risks, as it can be accompanied by adverse effects such as bradycardia, oxygen desaturation, and pulmonary hemorrhage. A liberal PS use policy not only increases the number of invasive procedures but also contributes to higher overall healthcare costs (In our local context, patient healthcare costs are covered through a combination of social medical insurance and out-of-pocket payments). Conversely, waiting for infants to reach the ventilator and FiO_2_ thresholds may delay surfactant administration and reduce its potential benefits. Consequently, a non-invasive, accurate strategy for early diagnosis of NRDS and guidance of PS therapy in preterm infants would be highly valuable.

Bedside lung ultrasound has become an important tool in diagnosing NRDS and can characterize different causes of infant respiratory distress accurately [1,7,8]. The lung ultrasound score (LUS) serves as a semi-quantitative measure to assess the severity of NRDS and predict the need for PS therapy. Lung ultrasound (LUS) findings have been well-established as correlating with the oxygen saturation to FiO_2_ ratio, enabling its application in accurately predicting the need for surfactant therapy in extremely preterm neonates with NRDS. [9,10,11,12]. Other studies have highlighted the advantages of lung ultrasound in reducing PS usage and decreasing radiation exposure in the neonatal intensive care unit (NICU) [12,13].

Despite its potential, the clinical application of LUS to guide PS therapy faces limitations. Firstly, no consensus exists on lung ultrasound scoring methods or LUS thresholds for PS administration. Secondly, some scholars argue that elevated LUS can also occur in non-NRDS pulmonary conditions, potentially limiting its accuracy in identifying infants who would benefit from PS [14]. Furthermore, there is a scarcity of randomized controlled trials focusing on LUS-guided PS administration.

In this study, we developed an early postnatal lung ultrasound assessment strategy suitable for very preterm infants (gestational age [GA] < 32 weeks), using lung ultrasound for diagnosis and LUS-guided PS administration. Through a randomized controlled trial, we aim to demonstrate that this strategy, compared to the conventional method (relying on clinical evaluation and FiO_2_), enables more precise and earlier surfactant administration, shortens the duration of mechanical ventilation, and reduces early radiation exposure.

## 2. Materials and Methods

### 2.1. Design

This study was a prospective, non-blinded, randomized controlled clinical trial conducted in the NICU of Peking University Third Hospital, Beijing, China, from August 2024 to May 2025. As a regional referral center for high-risk neonates and pregnant women, this parturition center has an annual delivery volume of over 6000 cases, including approximately 200 preterm infants (GA < 32 weeks) each year. The study was conducted in accordance with the Declaration of Helsinki and was reviewed and approved by the Medical Science Research Ethics Committee of Peking University Third Hospital (approval number: M2023337). Written informed consent was obtained from the legal guardians of all enrolled participants. This trial was registered at ClinicalTrials.gov (registration number: NCT06491901). The study protocol was published previously [15].

### 2.2. Infants

#### 2.2.1. Inclusion Criteria

Infants were eligible for inclusion if they met all of the following criteria: (1) preterm infants with GA < 32 weeks; (2) presence of postnatal respiratory distress, defined as a respiratory rate > 60 breaths/min, grunting, nasal flaring, intercostal retractions, and/or cyanosis; and (3) born at Peking University Third Hospital.

#### 2.2.2. Exclusion Criteria

Infants were excluded if they met any of the following criteria: (1) were outborn (i.e., delivered at another hospital), or received PS in the delivery room at our facility; (2) had known or confirmed congenital abnormalities identified during diagnosis or treatment, especially cardiopulmonary malformations; (3) had severe complications at birth, including severe asphyxia, hemorrhagic shock, pneumonia, pneumothorax, early-onset sepsis, or pleural effusion, or other diseases not caused by PS deficiency; (4) died within the first 72 h of postnatal age, were transferred to another hospital for surgery within 7 days after birth, or had incomplete data; or (5) whose families did not provide consent to participate in the study.

### 2.3. Objectives

The primary outcome measure for this study is the duration of mechanical ventilation during hospitalization in the ultrasound and control groups. Secondary outcome measures encompass (1) the utilization rate of PS in both groups; (2) the time from birth to the initial PS administration; (3) the times of radiation exposure in both groups (before PS, 7 day after birth); (4) the S/F (SiO_2_/FiO_2_) at 24 h and 72 h after birth; and (5) the incidence rates of BPD, intraventricular hemorrhage (IVH), retinopathy of prematurity (ROP), extrauterine growth restriction (EUGR), and necrotizing enterocolitis (NEC).

### 2.4. Methods

**Figure 1:**
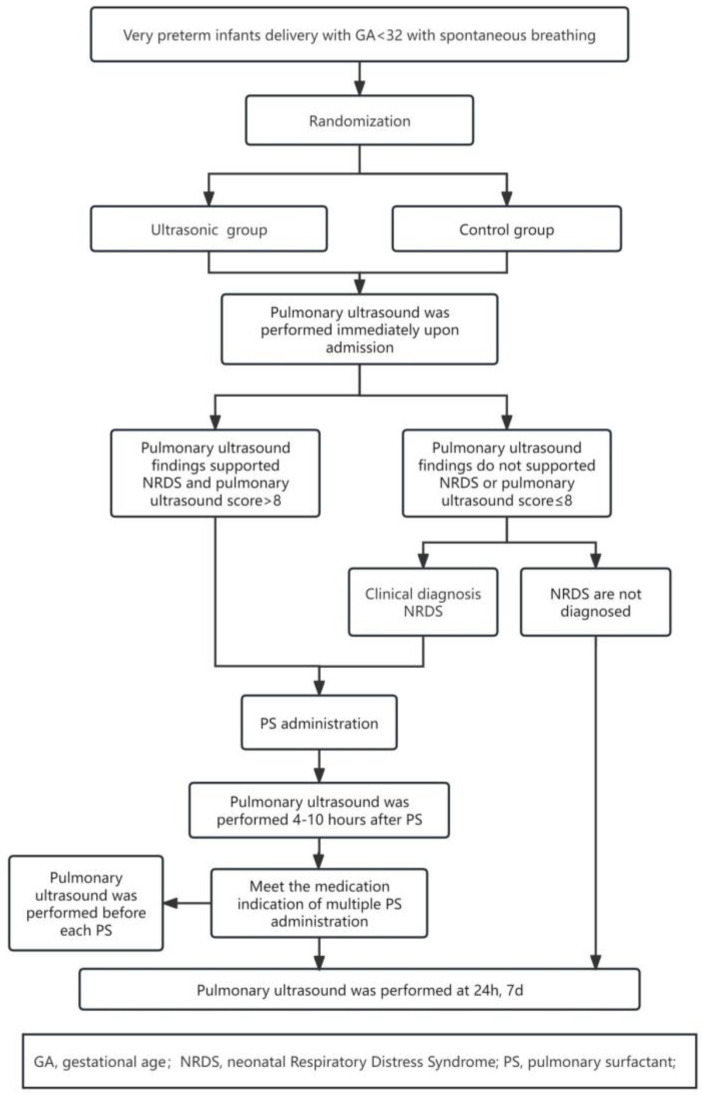
Flowchart of Infant Selection and Treatment.

Infants who met the inclusion criteria underwent standardized resuscitation procedures after birth in accordance with the European neonatal resuscitation guidelines [16] and were randomly assigned (1:1) to either the ultrasound group or the control group upon NICU admission. Randomization was performed using a predetermined random number table generated by an independent statistician with computer software (R, version 4.3.1), and group assignments were concealed in sealed, opaque envelopes. For multiple births, randomization was conducted separately for each infant. Lung ultrasound is a routine examination in this NICU. This study involved two ultrasound specialists and five neonatologists. Both ultrasound specialists received comprehensive training in lung ultrasound examination and the application of the LUS scoring system. Two ultrasound physicians, blinded to each other’s assessments, independently assessed the lung ultrasound scoring consistency. If the scores were consistent, the result was recorded. If there was a difference of ≥2 points, the final score was determined through discussion between the two ultrasound specialists. Enrolled infants followed the selection and treatment procedures shown in Figure 1.

#### 2.4.1. Ultrasound Group

Lung ultrasound was performed within 1 h of admission, followed by lung ultrasound scoring. If the lung ultrasound confirmed NRDS and LUS > 8, a full dose of PS at 200 mg/kg (Poractant Alfa, Curosurf^®^, Chiesi Farmaceutici, Parma, Italy) was promptly administered. The diagnostic criteria for NRDS based on lung ultrasound were derived from the 2019 guidelines authored by Jing Liu et al. [17]. The cutoff value of 8 was selected based on previous studies that adopted the same lung ultrasound scoring method [9,18]. If the lung ultrasound did not indicate RDS or the LUS ≤ 8, clinical monitoring was continued. If infants met the clinical diagnostic criteria for NRDS during monitoring, a full dose of PS (200 mg/kg, Poractant Alfa, Curosurf^®^, Chiesi Farmaceutici, Parma, Italy) was administered via the Intubate-Surfactant-Extubate (INSURE) technique for infants on non-invasive support, or via standard intratracheal instillation for those already intubated. Lung ultrasound and scoring were repeated 4–10 h after the initial PS dose, with scoring results withheld from the clinical team. Repeated PS administration was performed if the criteria were met, with lung ultrasound scoring conducted before each administration.

#### 2.4.2. Control Group

Infants in the control group also underwent lung ultrasound examination and scoring within the first hour of postnatal age; however, the results were not disclosed to the clinicians. If, during monitoring, infants met the clinical diagnostic criteria for NRDS (and FiO_2_ reached the threshold), PS was administered at a dose of 200 mg/kg (Poractant Alfa, Curosurf^®^, Chiesi Farmaceutici, Parma, Italy) via the Intubate-Surfactant-Extubate (INSURE) technique for infants on non-invasive support, or via standard intratracheal instillation for those already intubated. If the criteria for NRDS were not met during monitoring, no PS was administered.

### 2.5. General Infant Management

**Figure 2:**
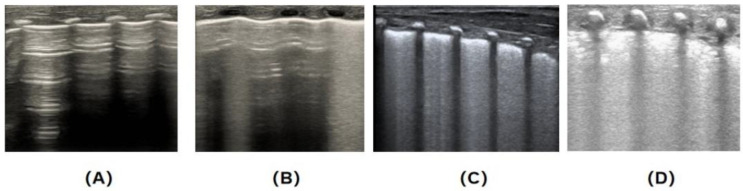
Calculation of Lung Ultrasound Score [15]. Segmentation: Each lung is divided into three regions—anterior (L12/R12), lateral (L34/R34), and posterior (L56/R56)—and both lungs comprise a total of six regions. The total score ranges from 0 to 18. A higher score indicates more severe lung involvement. The detailed scoring criteria are as follows: (**A**) **0 points (normal aeration):** Characterized by A-lines with sporadic B-lines; (**B**) **1 point (moderate aeration loss):** Including fused B-lines and interstitial syndrome (B-line occupying < 50% of the examined lung field); (**C**) **2 points (severe aeration loss):** With a small amount of localized subpleural consolidation (B-line occupying ≥ 50% of the examined lung field; B-line covers more than half of the intercostal space); (**D**) **3 points (extensive consolidation):** Extensive lung consolidation.

1. Resuscitation Procedures in the Delivery Room and Operating Room: Throughout ventilation and transportation, a T-piece resuscitator was used to provide either non-invasive or invasive respiratory support (initial parameters: PEEP = 6 cm H_2_O, PIP = 15–20 cm H_2_O, FiO_2_ ≤ 0.4) to maintain target oxygen saturation in infants. If the target oxygen saturation was not achieved, PEEP and FiO_2_ were upregulated; however, to avoid pneumothorax, PEEP was controlled at ≤8 cm H_2_O [1]. Our NICU implemented the “golden 1-h” management for premature infants [19,20]. Premature infants who successfully recovered were transferred to the NICU ward within 15 min and reached a relatively stable state shortly after birth.
2. Clinical Diagnostic Criteria for NRDS (1): Within the first 24 h of postnatal age, under invasive or non-invasive ventilation, the infant’s respiratory distress signs progressively worsen and meet one of the following criteria: (1) respiratory rate exceeding 60 breaths per minute, accompanied by nasal flaring, grunting, cyanosis, and other signs of respiratory distress; (2) peripheral capillary oxygen saturation (SpO_2_) ≤ 85% while FiO_2_ ≥ 0.4 (or ≥0.3 for infants with GA < 28 weeks); (3) Silverman-Anderson (SA) score > 5, or an hourly increase in SA score > 2; (4) with or without typical imaging changes. These signs may or may not be accompanied by typical chest X-ray findings.
3. Ultrasound Machine Settings: Lung ultrasound was performed using a Mindray M9 ultrasound system equipped with a linear, high-frequency (10–15 MHz) transducer. The lungs were divided according to the 6-region method described in the lung ultrasound guidelines.
4. The diagnostic criteria for NRDS based on lung ultrasound: (i) lung consolidations with air bronchograms, representing the most significant sign; (ii) an abnormal pleural line with absent A-lines; (iii) the potential presence of alveolar-interstitial syndrome (AIS) in non-consolidated areas; and (iv) unilateral or bilateral pleural effusion, observed in 15–20% of patients [17].
5. Lung ultrasound scoring method: Each lung was divided into three regions: anterior, lateral, and posterior. The anterior and posterior axillary lines were used as boundaries to divide each lung into anterior, lateral, and posterior regions. As a result, a total of six regions were defined for both lungs [17]. An ultrasound probe was placed perpendicular to the intercostal spaces to scan each region from top to bottom, and each region was scored. A score of 0–3, as proposed by Brat et al., was assigned to each region based on the ultrasound pattern detected [11]; if different patterns were observed within the same region, the highest (i.e., worst) score was recorded (Figure 2).

6. Indications for Repeated PS Administration: Within the first 72 h of postnatal age (i.e., 10–70 h after the initial PS administration), if respiratory distress persisted, chest X-ray findings confirmed NRDS, and other causes of respiratory distress were excluded. The dose for repeated PS administration was 100 mg/kg (Poractant Alfa, Curosurf^®^, Chiesi Farmaceutici, Parma, Italy).
7. Criteria for discontinuation of non-invasive ventilation: (1) >72 h of postnatal age; (2) PEEP ≤ 3–5 cm H_2_O during nasal continuous positive airway pressure (NCPAP), or mean airway pressure (MAP) < 6–7 cm H_2_O during nasal intermittent positive pressure ventilation (NIPPV); (3) FiO_2_ ≤ 25%; (4) SA score < 3; and (5) absence of respiratory pauses or bradycardia requiring stimulation for recovery. The criterion for discontinuing supplemental oxygen was maintenance of SpO_2_ > 90% for at least 3 days without supplemental oxygen.
8. The BPD diagnosis adhered to the 2018 National Institute of Child Health and Human Development (NICHD) classification. Diagnosis of hsPDA (hemodynamically significant patent ductus arteriosus) relied on clinical signs and echocardiographic findings [21]. IVH and white matter injury were diagnosed via cranial ultrasound, with IVH graded according to the Papile classification [22]. NEC (necrotizing enterocolitis) staging followed the modified Bell criteria [23]. ROP diagnosis and staging were based on fundoscopic examinations conducted by ophthalmologists.

### 2.6. Sample Size Calculation

Sample size calculation was based on the anticipated difference in the primary outcome (duration of invasive mechanical ventilation) between groups. Using departmental data (conventional group: 96 ± 12 h) and expecting a reduction to 86 ± 12 h with LUS guidance, a sample of 24 infants per group (48 total) was determined via PASS15 (two-sample t-test, α = 0.05, power = 80%). This was increased to 30 per group (60 total) to mitigate risks from effect size underestimation and dropouts.

### 2.7. Clinical Data Collection

Infant demographic data included GA, birth weight, gender, antenatal corticosteroid use, mode of delivery, intrauterine distress, and maternal pregnancy complications. Clinical data were collected at specified time points (at admission, pre-PS, 24 h, and 7 days after birth), including (1) timing, dosage, and method of the initial PS administration; (2) SA score, ventilation mode, oxygen saturation index, and FiO_2_ levels; and (3) LUS at each time point. (4) The duration of mechanical ventilation. Recorded complications and outcomes included (1) respiratory complications—occurrence and severity of infectious pneumonia, pneumothorax, pulmonary hemorrhage, and BPD; (2) cardiovascular complications—hemodynamically significant patent ductus arteriosus (hsPDA); (3) other complications in preterm infants, including IVH, periventricular leukomalacia, NEC, late-onset sepsis, ROP, EUGR, and others. The complications and other outcomes were set at either a corrected GA of 36 weeks or hospital discharge, whichever occurred earlier.

### 2.8. Statistical Analyses

Statistical analyses were conducted with SPSS 22.0. Continuous variables are presented as mean ± SD or median (IQR), and compared using the independent t-test or Mann–Whitney U test, as appropriate. Categorical variables are expressed as n (%), and compared using the χ^2^ test or Fisher’s exact test. A *p*-value < 0.05 was considered statistically significant.

## 3. Results

### 3.1. Baseline Clinical Characteristics

**Figure 3:**
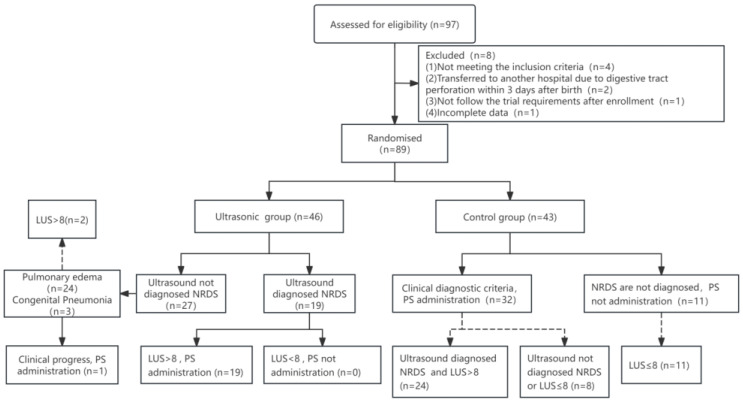
Flow Chart of Infants.

A total of 89 preterm infants with GA < 32 weeks were ultimately enrolled in the study. Among them, 46 were assigned to the ultrasound group and 43 to the control group. All infants were followed up according to the study protocol until hospital discharge or a corrected gestational age (CA) of 36 weeks (Figure 3). During the follow-up period, two infants from the ultrasound group and three from the control group were transferred to other hospitals after the 7th day of life for surgical reasons, resulting in incomplete outcome data for these participants.

In the ultrasound group (*n* = 46), based on lung ultrasound findings, 19 infants were diagnosed with NRDS and LUS > 8; all of them received PS administration. The remaining 27 infants were diagnosed with either pulmonary edema (*n* = 24) or congenital pneumonia (*n* = 3) based on lung ultrasound findings. Among these, two infants had an LUS > 8. Of the infants initially diagnosed with pulmonary edema, one developed worsening respiratory distress and increased oxygen requirement at 34 h after birth. This infant was subsequently considered to have developed ARDS secondary to PS inactivation and received PS administration. The other 26 infants did not receive PS administration. One infant in the ultrasound group (2.2%) required a second dose of PS, and no infant required a third dose.

**Figure 4:**
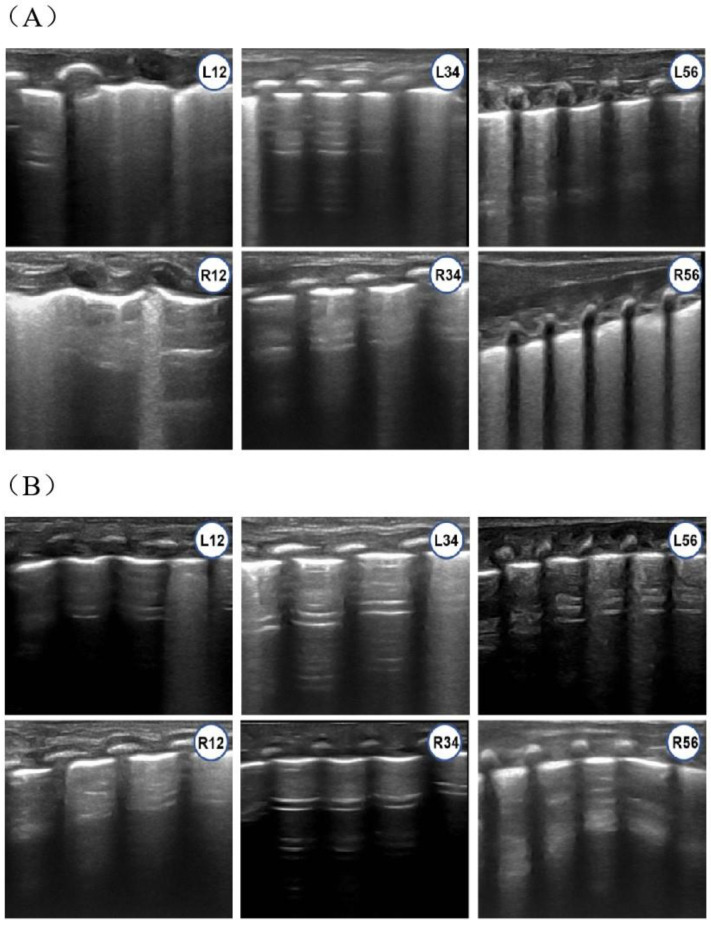
Lung Ultrasound Findings of an Infant with pulmonary edema (Lung Ultrasound Score = 9). (**A**) A preterm infant born at a gestational age of 31 + 5 weeks underwent lung ultrasound at 1 h of postnatal age. The findings showed both the disappearance of A-lines and the presence of an AIS (alveolar interstitial syndrome) pattern in regions L12, L56, R12, and R56. B-line occupying more than 50% of the lung fields in these regions, each lung fields LUS was 2. Regions L34 showed A-lines occupied more than 50% of the lung fields and AIS occupied two intercostal spaces, LUS was 1. Regions R34 showed A-lines in each intercostal spaces with sporadic B-lines, LUS was 0. No lung consolidation was observed in any lung field. Although the total LUS was 9, the findings were not consistent with NRDS. The diagnosis was pulmonary edema and PS was not administered. (**B**) Lung ultrasound re-examination at 24 h of postnatal age of this infant showed clear A-lines in all lung fields, a small number of B-lines, and only a few intercostal spaces with an AIS pattern. No lung consolidation was observed.

Figure 4 shows the lung ultrasound findings of an infant diagnosed with pulmonary edema, who had an LUS of 9.

In the control group (*n* = 43), 32 infants met the clinical diagnostic criteria for NRDS and received PS. Review of the ultrasound images collected before PS, eight of these infants did not meet the lung ultrasound diagnostic criteria for NRDS or had an LUS score ≤ 8. The remaining 11 infants in the control group did not receive PS; retrospective lung ultrasound review showed that none were diagnosed with NRDS. Three infants in the control group (7.0%) required a second dose of PS, and no infant required a third dose.

The baseline clinical characteristics of the two groups are shown in Table 1. There were no significant differences between the two groups in terms of GA, weight, rates of antenatal corticosteroid use, rates of antenatal antibiotic use, early NICU admission SA scores, or pre-surfactant S/F ratios.

### 3.2. Primary Outcomes

In the ultrasound group (*n* = 46), 9 infants (19.6%) received invasive mechanical ventilation, with a median duration of 0 h (IQR: 0–0) for the entire group. In the control group (*n* = 43), 20 infants (46.5%) received invasive ventilation, with a group median duration of 0 h (IQR: 0–86). The significant difference in the duration of invasive ventilation (Z = −2.81, *p* = 0.005) and proportion of infants requiring invasive ventilation (*p* = 0.007) reflects the substantial difference in the need for invasive respiratory support between the two groups.

The median total duration of respiratory support (including invasive and non-invasive ventilation) was 636.0 h (IQR: 490.5–859.0) in the ultrasound group and 620.0 h (IQR: 273.8–998.5) in the control group, with no statistically significant difference between the groups (Z = –0.65, *p* = 0.513).

### 3.3. Secondary Outcomes

In the ultrasound group, 20 out of 46 infants (43.5%) received PS administration, whereas in the control group, 32 out of 43 infants (74.4%) received PS administration. The proportion of infants receiving PS administration was significantly lower in the ultrasound group than in the control group (*p* = 0.003).

Excluding one infant in the ultrasound group who developed ARDS secondary to pulmonary edema and received PS at 34 h after birth, the median time to first PS administration in the ultrasound group (*n* = 19) was 60.0 min after birth (IQR: 48–87), which was significantly earlier than that in the control group (87.5 min; IQR: 63–123.5) (Z = –2.38, *p* = 0.017).

The number of X-ray exposures before PS administration and within 7 days after birth was significantly lower in the ultrasound group than in the control group (*p* = 0.023 and *p* = 0.019, respectively).

The results for other secondary outcomes are shown in Table 2 and Table 3. Regarding preterm infant complications, the incidence of hsPDA and EUGR was lower in the ultrasound group than in the control group (*p* = 0.018 and *p* = 0.031, respectively). No significant differences were observed between the two groups in the S/F ratio at 24 h and 7 days after birth, nor in the incidence of BPD, ROP, IVH (>II), or NEC.

## 4. Discussion

This randomized controlled trial demonstrates that an early postnatal lung ultrasound-guided strategy for the diagnosis of NRDS and surfactant administration in very preterm infants (GA < 32 weeks) is feasible and effective. Compared to the conventional approach based on clinical criteria and FiO_2_ thresholds, the LUS-guided strategy resulted in a more targeted use of surfactant, facilitated significantly earlier treatment in infants who required it, and reduced the need for invasive mechanical ventilation and radiation exposure in the early postnatal period.

In recent years, a growing body of research has explored the use of LUS to guide PS administration, aiming to establish reliable methods, optimal thresholds, appropriate timing, and associated clinical benefits [9,10,11,18]. However, several issues still limit the clinical application of LUS. For instance, different studies have employed inconsistent LUS methodologies and cutoff values for PS administration. Some studies have limited scoring to the anterior and lateral lung regions only [24,25]. Unlike chest X-ray findings, LUS manifestations and the severity of NRDS vary considerably across different lung zones. Due to gravitational effects, the posterior regions are more prone to alveolar collapse and fluid accumulation; therefore, omitting these areas may compromise diagnostic accuracy and LUS scoring reliability [26]. Furthermore, studies have shown that non-NRDS pulmonary conditions—such as pulmonary edema, pneumonia, and meconium aspiration syndrome—can also elevate LUS. As these conditions do not require PS therapy, guiding surfactant administration based solely on LUS may lack specificity [14]. To address these limitations, the present study implemented two main improvements based on previous studies. First, a comprehensive LUS assessment was performed in 12 lung regions (anterior, lateral, and posterior), and a total score was calculated to more accurately and thoroughly reflect pulmonary lesions in each infant. Second, a novel lung ultrasound-based strategy was developed—combining lung ultrasound diagnosis with LUS-guided PS administration—to minimize the influence of non-NRDS pulmonary diseases on treatment decisions. The detailed study protocol has been published previously [15].

A key finding of our study is the significant reduction in the rate of invasive mechanical ventilation achieved through the LUS-guided strategy. Most notably, the proportion of infants requiring any invasive ventilation was more than halved in the ultrasound group (19.6% vs. 46.5% in the control group). This represents a critical clinical outcome, as avoiding intubation and mechanical ventilation altogether is a primary objective in neonatology, directly mitigating the risks of ventilator-induced lung injury and other iatrogenic complications. We attribute this success to the optimized early respiratory management enabled by lung ultrasound. Numerous studies have demonstrated that early PS administration improves outcomes in NRDS, including shortening the duration of invasive ventilation, reducing the incidence of BPD, and lowering mortality [3,4,12,27]. For managing preterm infants with GA < 32 weeks during the early postnatal period, the “golden hour” strategy is recommended to rapidly achieve stabilization [20,28], which further challenges the timing of PS administration. In clinical practice, the FiO_2_ requirement in very preterm infants often manifests gradually, meaning that waiting for infants to reach the traditional FiO_2_ threshold for PS might delay optimal treatment timing. Existing studies have indicated that LUS predicts the need for PS in preterm infants more effectively than conventional clinical methods (such as SA score, FiO_2_, or X-ray) and aids in guiding early postnatal PS therapy [9,10,29]. In our study, the median time to first PS administration in the ultrasound group was 60 min after birth, significantly earlier than in the control group, consistent with previous findings. This confirms that LUS better reflects the severity of pulmonary pathology, enabling earlier PS administration and allowing more effective implementation of the “golden hour” strategy in preterm infants. The primary goal of early PS administration is to improve respiratory outcomes, such as the duration of mechanical ventilation and the incidence of BPD; however, randomized controlled trial research focusing specifically on this link remains scarce. In a previous quality improvement study, LUS-guided PS administration facilitated earlier PS use, subsequently shortening the duration of invasive ventilation and oxygen exposure [30]. Another randomized controlled trial also demonstrated a higher proportion of early PS administration (within 3 h) in the LUS-guided group compared to the control group, although that study found no difference in the duration of ventilation or the incidence of BPD [18]. Although the reduction in the duration of invasive ventilation was statistically significant in our findings, the absolute decrease observed was modest. For very preterm infants, the first 24–72 h represent a critical window during which lung injury is most likely to occur. Even a reduction of just several hours in mechanical ventilation during this vulnerable period can meaningfully alleviate the cumulative mechanical stress on the immature lungs. Postnatal oxygen therapy, mechanical ventilation, and volutrauma remain major contributors to BPD [31], and theoretically, reducing early invasive ventilation in preterm infants could lower the long-term incidence of BPD [32]. However, similar to previous studies, our findings did not demonstrate a significant advantage for the ultrasound group in reducing BPD incidence. This observation may be related to the relatively small sample size and the low overall incidence of BPD. Future studies with larger sample sizes are warranted.

Another conclusion of this study is that the pulmonary ultrasound strategy effectively reduces the use of early PS in very premature infants. Currently, the rate of PS use in preterm infants with GA < 32 weeks remains high (approximately 80–90%) in developing countries and regions [33]. Prophylactic PS administration is still chosen by many clinicians and is believed to improve outcomes, although the criteria for prophylactic use remain inconsistent [34]. However, studies have shown that the misdiagnosis rate for NRDS can be as high as 62–77% [5,35], and PS overuse increases the financial burden on families. Therefore, deciding on PS administration within a narrow time window remains a clinical challenge. Retrospective studies suggest that lung ultrasound can diagnose NRDS earlier and more intuitively and can reduce the rate of PS use by 30.1% [12]. In a similar study by Rodriguez-Fanjul et al., the probability of PS administration was significantly lower in the LUS-guided group than in the control group (41.3% vs. 62.9%) [18]. The lung ultrasound assessment protocol used in our study, which combines ultrasound diagnosis with LUS scoring, further restricted PS use. The results showed a significantly lower PS administration rate in the ultrasound group compared to the control group (43.5% vs. 74.4%), while the rate of repeated PS doses did not differ significantly between groups (2.2% vs. 7.0%). Among the infants in the ultrasound group who, after initial assessment, did not receive PS administration, all but one experienced relief from respiratory distress with ventilatory support alone; the one exception received PS administration at 34 h of postnatal age due to ARDS secondary to lung edema. A retrospective review of lung ultrasound images from the control group infants who received PS revealed that eight did not meet the ultrasound criteria for NRDS, suggesting that PS administration might have been avoidable in these cases. These findings collectively indicate that the lung ultrasound strategy employed not only reduced the rate of PS use but also did not delay necessary administration or increase the need for repeat doses. Instead, it was associated with reduced invasive ventilation, highlighting the clinical advantage of this strategy.

Regarding other complications in preterm infants, the results of this study indicate that the incidence of hsPDA and EUGR was lower in the ultrasound group than in the control group. However, the ultrasound group shows no advantage in terms of NEC, ROP, or grade II or higher IVH. To our knowledge, no previous studies have reported that LUS-guided PS administration can reduce the incidence of hsPDA and long-term EUGR. However, some studies have indicated that use of PS is a significant risk factor for the occurrence of hsPDA in premature infants aged 26–29 weeks [36]. Whether this can explain the lower hsPDA incidence observed in the ultrasound group requires further investigation. Similarly, previous research has shown that improved early oxygenation status and shorter duration of mechanical ventilation may help enhance the nutritional status of preterm infants during hospitalization. A multicenter study in the United States of very low birth weight infants indicated that postnatal weight at 2–4 weeks is associated with continued dependence on mechanical ventilation and with pulmonary dysfunction at 7 days of postnatal age [37]. Another study suggested that prolonged respiratory support and increased work of breathing can lead to insufficient early energy intake in preterm infants, resulting in a catabolic state and increased energy expenditure. This, in turn, delays the establishment of full enteral nutrition, slows weight gain, and ultimately increases the incidence of EUGR [38]. In the present study, both the rate and duration of invasive mechanical ventilation were significantly lower in the ultrasound group than in the control group, which may have contributed to improved early nutritional status and a subsequent reduction in EUGR.

Preterm infants with GA < 32 weeks are also at high risk for radiation exposure—a risk substantially greater than that for term infants. As a non-invasive imaging modality, lung ultrasound can effectively reduce early radiation exposure in this population [13,39]. In this study, infants in the ultrasound group no longer relied on chest X-rays for the diagnosis of NRDS or as a reference for PS administration. Consequently, the frequency of X-ray exposure before PS treatment and within the first 7 days after birth was significantly lower in the ultrasound group than in the control group. While the absolute reduction in the number of X-rays may appear modest, its clinical importance is substantial for this vulnerable population. Adhering to the ALARA (As Low As Reasonably Achievable) principle, every avoided exposure contributes to a lower lifetime cumulative radiation dose and associated cancer risk [39]. Therefore, a 50% reduction in median exposure within the critical first week of life represents a meaningful advancement in patient safety. These findings demonstrate that early LUS-guided PS administration can effectively reduce the risk of early postnatal radiation exposure in preterm infants with GA < 32 weeks.

However, this study has a few limitations. (1) This was a single-center study with a relatively small sample size. (2) This study was not double-blinded because blinding ultrasound specialists was not feasible. Both the ultrasound specialists and the neonatologists were aware of the group assignments prior to PS administration. Minimizing subjective bias in ultrasound scoring involved two specialists independently scoring each infant, with the average score subsequently used. In addition, except for the first ultrasound examination, subsequent ultrasound results were not disclosed to the neonatologists to avoid influencing further treatment decisions. (3) Since all study participants were preterm infants with GA < 32 weeks and blood sampling was minimized, oxygenation status was assessed with the SF ratio (SiO_2_/FiO_2_) rather than the more accurate PF ratio (PaO_2_/FiO_2_). (4) The current study’s endpoint was set at 36 weeks of corrected gestational age or discharge, and did not include follow-up on long-term outcomes after discharge, such as long-term pulmonary injury, readmission rates, and the incidence of recurrent respiratory infections [40]. Future studies should focus on further follow-up and investigation of these long-term outcomes. Therefore, future research should include large-scale, multicenter randomized trials and utilize more objective evaluation metrics.

## 5. Conclusions

This randomized controlled trial confirms the feasibility and effectiveness of an early postnatal lung ultrasound-guided strategy for diagnosing NRDS and guiding surfactant therapy in very preterm infants (GA < 32 weeks). Compared to conventional methods, this strategy approach enabled more targeted surfactant use, facilitated earlier treatment, and significantly reduced the need for invasive mechanical ventilation and early radiation exposure.

**Clinical Implications:** Implementing ultrasound-guided surfactant administration allows for more precise and timely intervention, supporting the “golden hour” stabilization goal while minimizing iatrogenic risks from ventilation and radiation.

**Public Health Implications:** By avoiding unnecessary surfactant use and invasive procedures, this strategy promotes efficient resource utilization. Reducing early lung injury may also improve long-term respiratory outcomes, potentially lowering the healthcare burden of bronchopulmonary dysplasia and related sequelae. Large-scale multicenter studies are needed to validate its cost-effectiveness and sustained benefits.

## Figures and Tables

**Table 1:** Comparison of Baseline Clinical Characteristics Between the 2 Groups.

	Ultrasound Group (*n* = 46)	Control Group (*n* = 43)	*p*
Male	20 (48.9)	21 (51.2)	0.612
GA (weeks)	29.7 ± 1.7	29.7 ± 1.8	0.900
Weight (grams)	1271.1 ± 287.8	1262.3 ± 338.0	0.895
Antenatal corticosteroids (complete course)	37(80.4)	32 (74.7)	0.497
Antenatal antibiotics	19 (41.3)	12 (27.9)	0.185
Caesarean section	34(73.9)	38 (88.4)	0.083
1 min Apgar score ≤ 7	7 (15.2)	2 (4.7)	0.089
SGA	3 (65.2)	5 (11.6)	0.398
NICU admission SA score	3.83 ± 1.4	4.16 ± 1.6	0.930
S/F before PS	296.1 ± 44.1	276.1 ± 54.5	0.084

Note: Values are expressed as mean ± SD, number (%). Abbreviations: GA, gestational age; S/F, SpO_2_/FiO_2_ ratio. SGA: small for gestational age.

**Table 2:** Summary of the Secondary Outcomes (Short-term outcomes).

	Ultrasound Group (*n* = 46)	Control Group (*n* = 43)	*p*
S/F at 24 h after PS	328.2 ± 35.9	320.3 ± 40.7	0.699
S/F at 7 days after PS	368.1 ± 42.7	369.9 ± 40.3	0.946
Total duration of oxygen therapy (h)	844.9 ± 383.8	873.7 ± 500.2	0.060
Number of X-ray exposures before PS (per infant)	0 [0, 0]	0 [0, 1]	0.023
Number of X-ray exposures at 7 days after birth (per infant)	1 [1, 2]	2 [1, 3]	0.019
Hs-PDA	4 (8.7)	12 (27.9)	0.018

Note: Values are expressed as mean ± SD, number (%), median [25th–75th centile]; Abbreviations: S/F, SpO_2_/FiO_2_ ratio.

**Table 3:** Summary of the Secondary Outcomes (Outcomes prior to discharge).

	Ultrasound Group	Control Group	*p*
BPD	17/44 (38.6)	14/40 (35.0)	0.730
ROP	8/44 (18.1)	12/40 (30.0)	0.204
IVH (>II)	3/44 (6.8)	4/40 (10.0)	0.912
EUGR	11/44 (25.0)	18/40 (45.0)	0.031
NEC	2/45 (44.4)	2/41 (48.8)	1.000

Note: number (%); Some long-term outcome indicators are missing.

## Data Availability

The raw data supporting the conclusions of this article will be made available by the authors, without undue reservation.

## Abbreviations

The following abbreviations are used in this manuscript:

AIS	Alveolar interstitial syndrome
CPAP	Continuous positive airway pressure
GA	Gestational age
IVH	Intraventricular hemorrhage
LUS	Lung ultrasound score
NICU	Neonatal intensive care unit
NRDS	Neonatal respiratory distress syndrome
PS	Pulmonary surfactant
ROP	Retinopathy of prematurity
SA	Silverman-Anderson
